# Nutrient intakes from a 24-hour recall survey among children aged 2–5 years, adolescent girls aged 10–18 years, and women aged 19–49 years in five regions of Niger

**DOI:** 10.7189/jogh.15.04247

**Published:** 2025-09-01

**Authors:** Issiak Balarabé Mahamane, Adamou Issa Ali, Yatta Théodore Almoustapha, Ag Bendech Mohamed, Moursi Mourad, Ntandou Bouzitou Gervais, Aboubacar Mahamadou, Annan Reginald

**Affiliations:** 1 National Information Platform for Nutrition, National Institute of Statistics, Niger; 2 Intake – Center for Dietary Assessment, FHI 360, Washington, DC, USA; 3 Food and Agriculture Organization, Niger Bureau, Niamey, Niger; 4 Ministry of Health, Niamey, Niger; 5 Kwame Nkrumah University of Science and Technology, Kumassi, Ghana

## Abstract

**Background:**

Conducting dietary intake surveys among vulnerable groups is essential for assessing interventions and informing decision-makers on progress in transforming food systems for supporting healthier diets. We aimed to describe the usual intakes of nutrients and their adequacy compared to estimated average requirements in three target groups; children aged 2–5 years, adolescent girls aged 10–18 years, and women aged 19–49 years in the regions of Dosso, Maradi, Tahoua, Tillabéri, and Zinder in Niger.

**Methods:**

We conducted the first quantitative food consumption survey in Niger. Specifically, we used 24-hour recalls to estimate foods consumed the previous day and carried out a repeat recall on 20% of the sample to model usual daily intakes using the National Cancer Institute method. We selected representative samples of each age group in the five administrative regions studied.

**Results:**

We included 1209 children aged 2–5 years, 1105 adolescent girls aged 10–18 years, and 1053 women aged 19–49 years. The median total energy intake was 1413 kcal (95% confidence interval (CI) = 1118, 1748) for children, 2227 kcal (95% CI = 1714, 2826) for adolescents, and 2552 kcal (95%CI = 1981, 3226) for women. The average proportion of energy from carbohydrates was above 69% for all target groups, while fat and protein accounted for just 20% and 10% in all groups, respectively. Animal protein accounted for only 11% of total protein intake in children, compared with 9.15% in adolescent girls and 9.78% in women. Usual calcium, vitamin B12, and vitamin A intakes were far below estimated average requirements, regardless of the target group or region.

**Conclusions:**

Considering the risk related to very high micronutrient deficiencies, particularly in calcium, vitamin A, and vitamin 12, urgent action needs to be taken in Niger through the accelerated implementation of the national roadmap on transforming local food systems for healthy diets.

Niger is a landlocked country with a surface area of 1 267 000 km^2^, two-thirds of which is desert, and a population of 26 million in 2024. It is classified as one of the poorest countries in the world, with an annual population growth rate of 3.9% [1]. Niger’s main economic activities are agriculture and livestock farming, which account for 40% of the country’s GDP. Rain-fed agriculture is widely practiced and is essential for producing the main staple food crops, including millet and sorghum. Groundnuts, cowpeas, yellow groundnuts, sesame, onions, and tiger nuts are the most widely grown cash crops in Niger. Some people grow off-season crops after the rainy season, including products such as potatoes, lettuce, and cabbage. Livestock activities are mainly raised by nomadic people and agropastoralists.

The prevalence of chronic hunger in Niger was 19.8% in 2021 [2]. According to a national nutrition survey carried out in 2022, the prevalence of stunting (low height for age) was high in 2022, at 47%, while acute malnutrition in children under five years of age was 12.2%, which is above the 10% alert threshold set by the World Health Organization (WHO); meanwhile, the prevalence of severe acute malnutrition was 2.4% [3] and remains above the WHO's critical thresholds [4]. The prevalence of anaemia was 72% in children aged 6–59 months in 2022 [5].

As indicated in the Fill the Nutrient Gap report [6], the fight against malnutrition in Niger is slowed down by problems of economic access to healthy food (safe and nutritious), chronic food insecurity linked to the seasonal effects of low population resilience, poor access to social safety nets, and other social aspects. Apart from years of relative food abundance, the availability of food energy has always been lower than the population's energy needs. There is also wide disparity in fruit and vegetable consumption between urban (more favoured) and rural areas, and between regions [7].

Longitudinal studies and quantitative surveys of individual and collective food consumption have not yet been carried out in Niger, posing a major limitation in planning programmes and monitoring changes in dietary patterns. For this reason, we undertook an initial survey to provide estimates of quantitative nutritional intakes for three vulnerable groups. Here we describe the usual intakes of nutrients and their adequacy in relation to recommended daily requirements in children aged 2–5 years, adolescent girls aged 10–18 years, and women aged 19–49 years in five regions with high levels of stunting in children under five years of age.

## METHODS

### Subjects

The study focusses on the quantitative food consumption by 24-hour recall of three specific target groups that represent the groups most vulnerable to malnutrition: children aged 2–5 years; adolescent girls aged 10–18 years; women aged 19–49 years (including pregnant and breastfeeding women). Our sample came from five of Niger's eight regions, which were the most affected by stunting among children under five years of age: Dosso (38.6%), Maradi (61.7%), Tahoua (43.1%), Tillabéri (41.8%), and Zinder (51.5%). We did not consider the Niamey and Agadez regions, with stunting prevalence rates of 18.5% and 35.5%, respectively. We also did not consider the Diffa region, despite it having a prevalence of stunting of 42.2%, given the context of insecurity linked to the Boko Haram nebula. Representative samples of the three groups were selected in each of the five administrative regions considered as strata. Details of the study methodology can be found elsewhere [8]. 

### Data collection

Data were collected between November and December 2019, a post-harvest period of relative food abundance in a year of good rainfall. In detail, we carried out this research in three phases, with two related to data collection and one to data analysis.

The first phase consisted of planning, including an inventory of foods and recipes commonly consumed in the five study regions, whereby we standardised 3129 recipes found in households and restaurants to obtain a database containing 428 recipes, each with its constituent ingredients, as well as conversion factors, cooking time, and cooking method. Once the food lists and standard recipe lists had been drawn up and a food composition table compiled, we conducted a pilot survey to test the quality of the planning and ensure corrective action on all aspects of the survey.

The second phase involved collecting food consumption data for the three target groups in these five regions using the 24-hour recall approach. This involved 32 clusters in each of the five study regions, with 20 households surveyed in each cluster. Also, 20% of those interviewed during the first recall were given a second 24-hour recall within 3–10 days of the first recall, allowing us to estimate the intra-personal variation in dietary intakes in the population, which can be accounted for when analysing the data in order to estimate a distribution of usual intakes in the population that reflects only the interpersonal variation. This database contains, among other things, information on the age and weight of individuals, as well as on the quantities, time of consumption within a given day, and method of measurement or estimation of food quantities consumed and recipe compositions.

### Sampling

We calculated our sample size based on the recommendations contained in the Fortification Rapid Assessment Tool (FRAT) study guides [9] and with the support of the INTAKE Center for Dietary Assessment [10], which specialises in food consumption surveys. This size was based on a confidence level of 95%, a precision (margin of error) of 10%, an expected prevalence of minimum consumption of the potential food vector of 50%, and a design effect of 2 [11]. Given the survey objectives and its coupling with the 24-hour recall food survey, we reconsidered the sample size to improve precision in relation to the FRAT guide recommendations. We had to sample 1275 individuals in each target group for the sample to be representative (Table S1 in the **Online Supplementary Document**).

We chose the household as the sampling unit. Specifically, we sampled a maximum of one individual within a household at random per target group to reduce the clustering effect. We introduced a skip sampling model as a means of accommodating multiple possible samples per household to reduce the response weight per household (Table S2 in the **Online Supplementary Document**). This method was prepared by a Professor of the Harvard School of Public Health for the purpose of this study [12].

Recall, generally based on the chronological order of the previous day’s food intake, is affected by defects in a respondent’s memory [13]. To address this, we used a technique developed in the USA to improve the quality of recall and limit under-reporting by respondents [14]. 

### Data processing and analysis

The third phase involved the creation and management of databases, including data processing and analysis. For this purpose, we used the median imputation method, as the median value is less sensitive to variations than the mean value. To avoid introducing bias into the treatment of outliers, the treatment was carried out independently for the three target groups (children aged 2–5, adolescents aged 15–18, and women aged 19–49).

As the Kolmogorov test for normality of residuals and the Bartlett test homogeneity of variances failed, we used the non-parametric Kruskal-Wallis test to explore overall variations in energy intake between certain regions and across all target groups. We then used Dunn’s test to determine variations between pairs of regions. We used a significance level of 5% throughout the study. These analyses were done in Stata, version 16.1 (College Station, TX, USA).

Niger does not have estimated average nutrient requirements at national level. For this reason, we estimated the average requirements at world level for each of the micronutrients selected (iron, zinc, folate, calcium, vitamin A, vitamin B6, and vitamin B12) within SAS, version 9.4 (Cary, NC, USA) prepared by the INTAKE Center for Dietary Assessment team using the harmonised average requirements (HAR) estimated at world level [11]. This programme enables the measurement of the adequacy of intakes of each of the micronutrients selected with the estimated average requirements considered. For all analyses of usual dietary intakes, we used the National Cancer Institute (NCI) [11], which utilises statistical modelling to estimate intrapersonal variation in food and nutrient intakes based on information from people who have undergone a first and second recall. The approach estimates a distribution of intakes for the whole population or sub-population of interest, which represents only the inter-personal variation.

In detail, we carried out the modelling of usual nutrient intakes using the NCI method based on the average nutrient requirements published for each age group [8]. For adolescents and women, average requirements were assigned according to status (pregnant, breastfeeding, non-pregnant, and non-breastfeeding). For zinc, the average requirements estimated for an unrefined diet were used for adult women, while the average requirements estimated for a semi-refined diet were applied for children and adolescents because this was the only option available. For iron, we adopted a low to moderate intake (7.5%).

We used the NCI method to estimate the percentage of the population whose intakes were below the estimated average requirements (cut-off point method). This method was applied for proteins and micronutrients, except for iron, which was assessed using the probability method, as iron requirements are asymmetrical. We utilised the iron module of the INTAKE Program for Usual Diet Assessment to estimate the distribution of usual iron intakes and assign probabilities of inadequacy to each intake in the distribution, after which we estimated the average probability over all individuals, which corresponds to the prevalence of inadequate iron intakes.

## RESULTS

We collected and analysed data on 1209 children aged 2–5 years, 1105 adolescent girls aged 10–18 years, and 1053 women aged 19–49 years across all five regions.

### Usual energy intake and proportion of energy from carbohydrates, fats. and proteins

**Figure 1:**
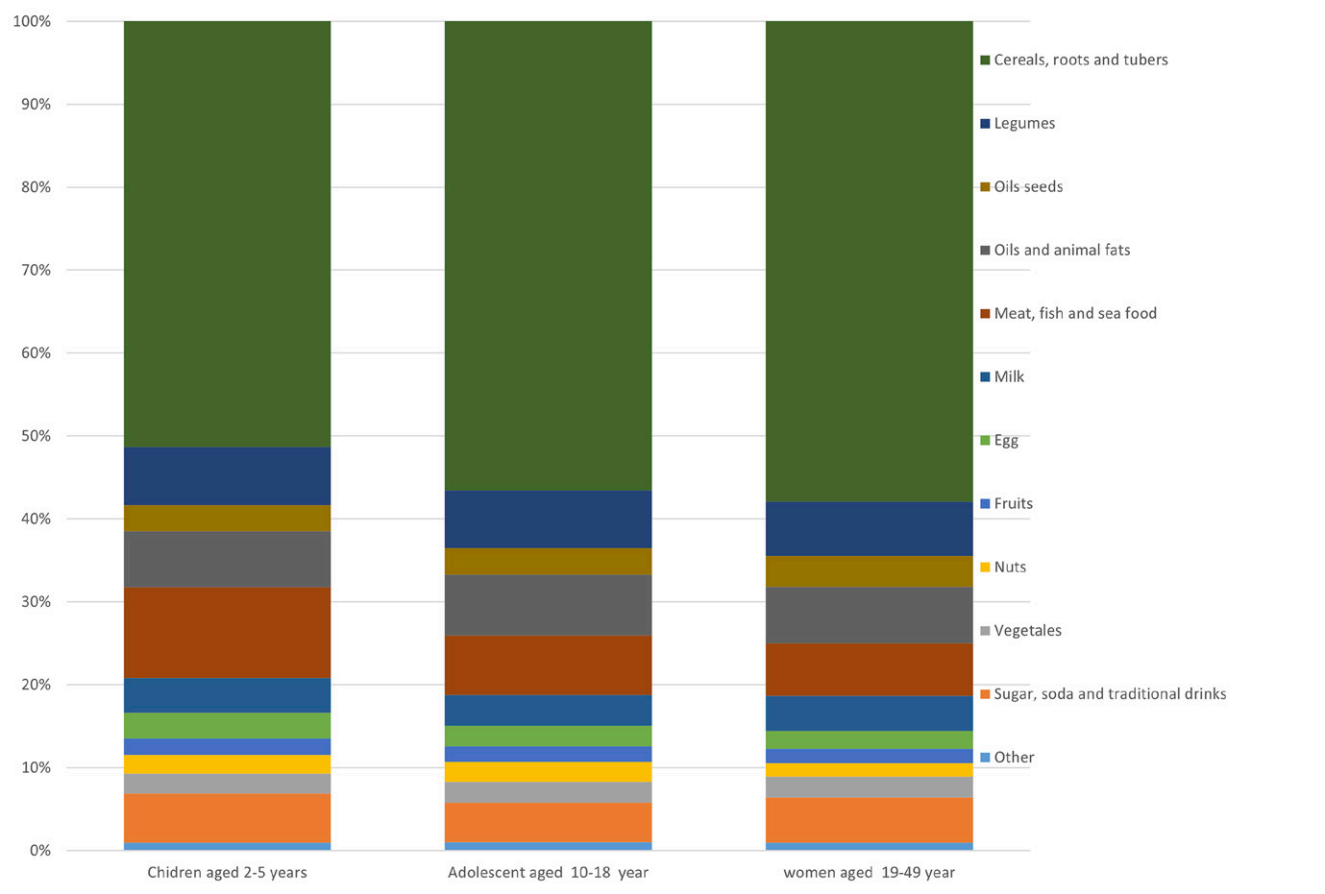
Share of energy intake from food groups consumed by the three vulnerable groups in all five study regions, by food group.

The average usual energy intakes were lower in the Maradi and Zinder regions than in the Dosso, Tahoua, and Tillabéri regions, except for children aged 2–5 years in the Tillabéri region (**Table 1**). The share of energy from the different food groups was dominated by cereals, roots, and tubers, accounting for 58%, 57%, and 51%, respectively, among women aged 19–49 years, adolescent girls aged 10–18 years, and children aged 2–5 years. Legumes and vegetable oils and animal fats each account for 7% of dietary energy, whatever the target group, while sugars, soft drinks and traditional beverages account for <7% (**Figure 1**). The average proportion of energy from carbohydrates (sugar and starch, mainly from cereals in Niger) was over 69% in all three groups and in all the regions studied, while the proportions from fat and protein were barely 20% and 10%, respectively (**Table 2**). The average usual protein intake was 38.40 g/d for children, compared with 60.10 g/d for teenage girls and 68.50 g/d for women. Animal protein accounts for 11.19% of total protein intake in children, compared with 9.15% in teenage girls and 9.78% in women. Among children, for example, there was a statistically significant difference in terms of habitual protein and lipid intake between the Dosso and Maradi regions. There were also statistically significant differences in usual protein, fat, and carbohydrate intake between the Tillabéri and Zinder regions. Between the pairs of regions (Tahoua and Dosso), there was no statistically significant difference in terms of macronutrient intake across all target groups (Table S3 in the **Online Supplementary Document**).

**Table 1:** Mean usual dietary energy intakes expressed in kcal for children aged 2–5 years, adolescent girls aged 10–18 years, and women aged 19–49 years, in all regions taken together and by study region

Nutrients	Region	Children aged 2–5	Teenage girls aged 10–18	Women aged 19–49
Energy (kcal)	All regions taken together	1 457 (31)	2 322 (52)	2 660 (60)
	Dosso	1 638 (65)	2 637 (145)	3 403 (117)
	Maradi	1 430 (63)	2 064 (102)	2 424 (129)
	Tahoua	1 675 (89)	3 123 (108)	3 204 (136)
	Tillabéri	1 393 (47)	2 346 (126)	2 832 (104)
	Zinder	1 193 (39)	1 582 (67)	1 860 (60

*Presented as mean (standard deviation).

**Table 2:** Proportion of energy from macronutrients and usual protein intake among children aged 2–5, adolescent girls aged 10–18, and women aged 19–49 in the regions taken together

Nutrients	Indicators	Children aged 2–5 (n = 1209)	Teenage girls aged 10–18 (n = 1105)	Women aged 19–49 (n = 1053)
Energy	Kcal/d, x̄ (SD)	1457 (31)	2322 (52)	2660 (60)
	% of energy from carbohydrates (95% CI at 5% risk)	69.69 (67.33, 70.44)	69.71 (67.13, 70.74)	71.18 (69.45, 71.96)
	% of energy from proteins (95% CI at 5% risk)	10.53 (10.31, 10.74)	10.47 (10.17, 10.76)	10.30 (10.03, 10.60)
	% of energy from lipids (95% CI at 5% risk)	19.78 (19.15, 20.42)	19.82 (18.93, 20.71)	18.52 (17.81, 19.24)
Proteins	Usual intake of proteins (g/d), x̄ (SD)	38.4 (0.92)	60.1 (1.59)	68.5 (1.72)
	% of the population whose usual intake is below the recommended daily requirement (95% CI at 5% risk)	0.20 (0.15, 0.56)	11.58 (5.94, 17.22)	19.30 (10.56, 28.04)
	Share (%) of animal products in total intake	11.19	9.15	9.78

CI – confidence interval, SD – standard deviation, x̄ – mean

### Vitamin and mineral inadequacy

In the five regions taken together, only 8.69% of adolescent girls aged 10–18 years cover their calcium needs through diet, compared with 18.92% of women aged 19–49 years and 27.76% of children aged 2–5 years (**Table 3**). The Maradi and Zinder regions have even lower coverage rates for all targeted age groups. Only 3.99% of women aged 19–49 years cover their vitamin A needs through food, compared with 7.31% of adolescent girls aged 10–18 years and 18.28% of children aged 2–5 years. The Maradi and Zinder regions have even lower coverage rates for all target groups. Similarly, only 11.10% of women aged between 19 and 49 years cover their needs through food, compared with 18.68% of children aged between 2–5 years and 14.50% of adolescent girls. The regions of Maradi, Zinder, and Dosso had even lower coverage rates.

**Table 3:** Usual intakes of vitamins A, B12, and calcium and their levels of adequacy in children aged 2–5 years, adolescents aged 10–18 years, and women aged 19–49 years in the regions taken together

Nutrients	Indicators	Children aged 2–5 (n = 1209)	Teenage girls aged 10–18 (n = 1105)	Women aged 19–49 (n = 1053)
Vitamin A (REA)	Intake (µg/d), x̄ (SD)	141 (12)	208 (18)	256 (20)
	% of the population with usual intakes below the recommended daily Requirement (95% CI at 5% risk)	81.72 (74.96, 88.48)	92.69 (88.21, 97.17)	96.01(91.49, 100.55)
Vitamin B12	Intake (µg/d), x̄ (SD)	0.67 (0.12)	0.93 (0.15)	1.04 (0.17)
	% of the population with usual intakes below the recommended daily requirement (95% CI at 5% risk)	81.32 (76.76, 85.89)	85.50 (80.62, 90.40)	88.90 (85.08, 92.72)
Calcium	Intake (mg/d) (EC	367 (11.36)	540 (16.78)	608 (18.11)
	% of the population with usual intakes below the recommended daily requirement (95% CI at 5% risk)	72.24 (68.16, 76.32)	91.31 (87.76, 94.85)	81.08 (73.79, 88.36)
Vitamin B6	Usual intake (µg/d), x̄ (SD)	1.17 (0.02)	1.86 (0.04)	2.16 (0.05)
	% of the population with usual intakes below the recommended daily requirement (95% CI at 5% risk)	3.35 (0.93, 5.78)	18.13 (12.33, 23.93)	13.60 (5.98, 21.22)
Folates	Intake (µg/d), x̄ (SD)	273 (8.67)	421 (14.08)	466 (14.31)
	% of the population with usual intakes below the recommended daily requirement (95% CI at 5% risk)	1.50 (−0.09, 3.09)	14.74 (9.52, 19.97)	24.02 (14.07, 33.98)
Iron	Intake (mg/d), x̄ (SD)	23 (0.55)	37 (1.00)	42 (1.00)
	% of the population with usual intakes below the recommended daily requirement (95% CI at 5% risk)	3.04 (1.76, 4.33)	5.23 (2.62, 7.83)	4.99 (2.51, 7.47)
Zinc	Intake (mg/d), x̄ (SD)	8.28 (0.19)	13.25 (0.34)	15.43 (0.37)
	% of the population with usual intakes below the recommended daily requirement (95% CI at 5% risk)	4.64 (2.35, 6.93)	24.46 (18.43, 30.50)	27.11 (19.44, 34.78)

CI – confidence interval, SD – standard deviation, x̄ – mean

In the five regions taken together, almost all children aged 2–5 years meet their folate (98.50%) and zinc (95.36%) requirements. Conversely, adolescent girls in all regions combined meet 85.26% and 75.54% of their daily folate and zinc requirements, respectively, while those living in the Maradi and Zinder regions have lower folate and zinc coverage than those in the other regions. Trends identical to those for adolescent girls were observed among women aged 19–49. Daily iron intakes were still well in excess of recommended requirements for almost the entire population, whatever the target group.

## DISCUSSION

### Average usual energy intake by study region

Taken together, the energy intakes among the three target groups in the five regions of Niger were close to the recommended daily intakes for the various groups studied. This relatively good result could be explained by the survey data collection period (November and December) corresponding to the post-harvest period, when cereals were more widely available in granaries and food prices were low on local markets. Among the cereals consumed in Niger, millet is the staple food most frequently consumed almost seven days a week and in large quantities by all the target groups in the five regions [15]. As 2019 was a good year in terms of rainfall and the agricultural season, there were generally no restrictions on the use of cereal staples. However, a reduction in the quantities of cereals consumed at the family level can be observed as we approach the lean season or period of low food availability, which peaks between May and September each year. The decision to collect data during the post-harvest period assumes that the risk of deficiencies is lower than during the lean period when food availability is low. Indeed, the levels of coverage/non-coverage of nutrient intakes observed in November and December would deteriorate further during the lean season, with even higher risks of non-coverage of needs and deficiencies in the Niger context.

The Maradi and Zinder regions are areas of intense food production and are the economic hubs of Niger. In Maradi, for example, 91% of the women surveyed earn money, almost half of them through petty trading [16]. They are rarely supported in these income-generating activities by their immediate social environment. The women’s modest additional resources (only 3% make a weekly profit of FCFA 5000 (USD 8.4) or more from trading) are spent in most cases on buying food for their children, themselves, and the family [16]. The regions of Maradi, Zinder, and Dosso have the widest gender gaps in access to education in Niger. The proportion of women aged 15–49 years with incomplete secondary education is particularly low in Maradi and Zinder, at 10.8% and 9.7%, respectively, compared with a national average of 15.2% [17]. The proportions of individuals who had used the internet in the 12 months preceding the survey were 5.6% and 4.4%, respectively, in Maradi and Zinder, compared with a national average of 11.1%. Practices such as child marriage and unfavourable learning environments are among the barriers to girls’ access to education in Niger [18]. Health coverage does not exceed 50% nationally, and the contribution of the community level is limited in the regions of Maradi and Zinder [19].

Poor access to adequate energy intake in these two regions could be explained by social, cultural, and behavioural barriers and poor access to basic social services, as well as by food availability at household and market levels. In the Maradi region, qualitative research revealed several constraints, including the monotony of family meals, dietary restrictions for children, pregnant women, and adolescents, and the voluntary spacing of meals for children to prepare them for the difficult living conditions and physical activities in rural areas, and to withstand hunger in the event of a food shortage [19].

Added to this is the coexistence of several circles of influence around the mother and child, and the strong survival of social practices and rites specific to each ethnic group beyond the administrative region, with numerous interferences that delay the expected positive transformations in child care and feeding [19]. Per our observations, the acquisition of knowledge and the willingness of mothers alone were not enough to bring about a massive change in favour of the adoption of nutrition messages conveyed by local health services, and the national communication strategies implemented have had a limited impact on reducing the many barriers to the adoption of good infant and young child feeding practices and the treatment of malnutrition.

### Coverage of micronutrient requirements and health outlook

The coverage of daily needs for calcium, vitamin A, and vitamin B12 is dramatically low in the five regions overall, irrespective of the age group considered. This very worrying risk of calcium and vitamin A and B12 deficiencies is corroborated by the Food and Agriculture Organization (FAO) estimates made in 2019, which showed that 92.4% of the population of Niger is unable to eat healthily [20]. In other words, only 7.4% of the population can eat healthily. Consumption and the proportion of the budget allocated to fruit and vegetables were important indicators of the affordability of a healthy diet. According to FAO estimates, the cost of a healthy diet (PPP in USD per capita per day) is 2.85 (FCFA 1710), showing how expensive healthy food is in relation to purchasing power in Niger.

Seasonal variations in the availability and price of food products are very marked between the postharvest season and the lean season (low food availability, particularly in terms of fresh produce). During the lean season, it is likely that calcium, vitamin B12, and vitamin A requirements will be even lower, with an extremely high risk of severe deficiencies. The probable risk of calcium and vitamin B12 deficiencies in all the groups studied throughout the year is justified, among other factors, by very poor access to animal products, which were their main sources, and the absence of specific dedicated programmes at national and local levels. The low coverage of vitamin A needs in children aged 2–5 years, meanwhile, would be compensated for by preventive vitamin A supplementation programmes, which have been offering vitamin A capsules twice a year to children aged 6–59 months for several decades, as well as vitamin A fortification of cooking oils [18] reaching the whole population, particularly in urban areas. Coverage of vitamin A supplementation for children aged 6–59 months has been estimated at 86% in 2019 at the national level [21]. In Niger, cooking oils (groundnut oil, palm oil, and palm kernel oil) are among the seven food products covered by standards and implementing decrees for micronutrient fortification.

The results of our survey showing inadequate intakes of several micronutrients are in line with most food consumption studies in West Africa [22–24]. In fact, studies conducted over the last 15 years reveal low intakes of vitamin A, vitamin B12, calcium, and zinc in children under five years of age, adolescents (10–18 years), and adult women. Studies based on 24-hour dietary recalls show that most populations do not reach the recommended daily intake, with particularly pronounced deficiencies in rural areas. Average vitamin A intakes vary between 283–375 μg RE/d in children and 382–475 μg RE/d in adult women; vitamin B12 intakes are generally less than 1 μg/d in children and rarely more than 1.3 μg/d in adults; calcium intake varies between 290–380 mg/d in children and 350–500 mg/d in women; while zinc intake remains between 3.2–3.8 mg/d in children and 5.37.8 mg/d in adult women.

Despite some modest improvements in urban areas and in countries that have implemented food fortification programmes (notably Ghana [25] and Nigeria [26]), overall trends show stagnation in rural areas and widening socioeconomic disparities. Regional crises (Ebola outbreak, COVID-19 pandemic, insecurity in the Sahel region) have also negatively impacted nutritional intakes, particularly in Burkina Faso [27,28], Mali, and Niger. Explanatory factors include limited dietary diversification, the prohibitive cost of micronutrient-rich foods, geographical disparities, and vulnerability to climatic and economic shocks.

During the survey, information on the use of multivitamin solutions, nutritional supplements, and ready-to-use therapeutic foods for the treatment of acute malnutrition was only noted when the respondent mentioned them with concrete evidence. Not all these preventive or curative industrial medicinal products were included in the estimate of nutritional intake because of the difficulty of systematising responses in a context of high illiteracy, which have been encountered in other contexts and communities with much higher literacy rates than Niger [29].

In the five regions together, almost all children aged 2–5 years met their folate (99.91%) and zinc (95%) requirements. On the other hand, adolescent girls in all regions combined met 85% and 75% of their daily folate and zinc requirements, respectively. These high levels of folate and zinc coverage for all target groups, particularly children, are surprising and unexpected in the context of the study.

Daily iron intakes were still well above the recommended daily intake for all target groups and regions, a result that contrasts with the high prevalence of iron deficiency [30] and anaemia [31] among the various survey targets. The prevalence of anaemia in children under the age of five varied across the five study regions between 69.7% in Tahoua and 57.2% in Tillabéri in 2019 [31]. This surprising result was also observed in a 24-hour recall food survey conducted in urban Bamako in 1994 [32]. Preventive programmes to combat anaemia and iron deficiency, in particular fortification and iron and folate supplementation, are still fragmented and localised, with poor geographical coverage [18]. Frequent infections in the various target groups surveyed undoubtedly play a role in reducing the absorption of ingested iron. However, this discrepancy is probably linked to the selection of certain food products and their iron content in the African food composition tables, such as millet, beans/cowpea, and green leaves, whose iron content is reduced to a very large extent during processing into flour and preparation of meals [33].

### Limitations of the study and corrective measures taken

This was a cross-sectional survey carried out during a period of food abundance with a view to assessing dietary diversity and the adequacy of nutritional intakes for each target group. Ideally, information should have been collected at well-defined time intervals, given the seasonal nature of certain products, but due to budgetary constraints, this study was carried out over a single period. In relation to our use of the 24-hour recall period, we note that a two-pass study (post-harvest and lean seasons) in pre-school children and the elderly compared nutrient intakes estimated by 24-hour recall and by recording food systematically weighed over three days in a rural area of Kenya showed that the levels of nutrient intake measured by the two techniques were similar [34]. However, a single recall was insufficient to meet the objectives of our study due to the daily variability of food intake, which is why, in the absence of multiple recalls, we carried out a second recall on 20% of the samples from each of the three target groups in each study region.

The main limitations associated with 24-hour recall are its reliance on the respondent's memory, both to identify the foods and beverages consumed and to estimate the individual quantities consumed of these foods [35]. These limitations were considered during the three main phases of our food survey in Niger. The planning was a long and meticulous process of learning and national capacity building, as this was the first experience of this type of complex and technically difficult survey. Experiences in planning this type of survey, documented by Intake and the FAO worldwide, were adapted to the Niger context. The recruitment criteria for the interviewers and supervisors took into account all the dialects spoken in the study regions in order to avoid language barriers in communities where French, the official language, is little spoken [36].

## CONCLUSIONS

Calcium and vitamin A and B12 intakes were deficient compared with recommended daily intakes in all three target groups, with calcium deficiencies more marked among adolescent girls and vitamin A and B12 deficiencies more marked among women aged 19–49 years, with Maradi and Zinder more severely affected.

Urgent action focussed on the production and consumption of micronutrient-rich foods is needed to address this issue. To this end, Niger has established a roadmap for transforming sustainable food systems [37] and the National Nutritional Security Policy 2017–2025 [38]. Accelerating the efficient implementation of these two strategic documents in a synergistic and complementary manner will undoubtedly contribute to improving nutritional intakes among the vulnerable groups studied. Social and behavioural change interventions accompanying programmes for access to culturally adapted and diversified diets are priorities highlighted in these policy documents. In terms of future research, we suggest that the same survey be repeated during the season of low food availability or ‘hunger gap’ to assess changes in nutritional intakes among the same vulnerable groups depending on the season.

## Additional material


Online Supplementary Document
