# Supplementary material for: Nutrient intakes from a 24-hour recall survey among children aged 2–5 years, adolescent girls aged 10–18 years, and women aged 19–49 years in five regions of Niger
Source: J Glob Health. 2025 Sep 1;15:04247. doi: 10.7189/jogh.15.04247 (PMC12400888; doi:10.7189/jogh.15.04247)
Supplement: Online Supplementary Document [file jogh-15-04247-s001.pdf]

Supplement to: Mahamane IB, Ali AI, Almoustapha YT, Mohamed AB, Mourad M, Gervais NB, Mahamadou A, Reginald A. Nutrient intakes from a 24-hour recall survey among children aged 2–5 years, adolescent girls aged 10–18 years and women aged 19–49 years in five regions of Niger. J Glob Health. 2025;15:04247.

Table S1. Sample size for target groups

Target Group	Number of targets per stratum	Number of strata	Number of targets for 5 strata
Children aged 24 to 59 months	255	5	1 275
Teenage girls aged 10–18 year	255	5	1 275
Women aged 19–49 year	255	5	1 275
Total	765		3 825

Table S2. Sampling skip

Househo ld	Children 24–59 months	Teenage girls aged 10–18	Women aged 19–49
1		Yes	Yes
2	Yes	Yes	
3	Yes	Yes	Yes
4		YES	
5	Yes	Yes	Yes
6	Yes	Yes	
7	Yes	Yes	Yes
8	Yes	Yes	
9	Yes	Yes	Yes
10	Yes	Yes	
11	Yes	Yes	Yes
12	Yes	Yes	
13	Yes	Yes	Yes
14	Yes	Yes	
15	Yes	Yes	Yes
16	Yes	Yes	
17	Yes	Yes	Yes
18	Yes	Yes	
19	Yes	Yes	Yes
20		Yes	Yes
Total	17	20	11

Table S3. Inter-regional variations in usual macronutrient intakes in children aged 2–5, teenage girls aged 10–18 and women aged 19–49

Targets	Regions	Dosso	Maradi	Tahoua	Tillabéri
Children	Maradi	α β			
	Tahoua		α Ω		
	Tillabéri	α Ω		α Ω	
	Zinder	α β Ω	α Ω	α β Ω	α Ω
Teenage girls	Maradi	α β Ω			
	Tahoua		α β Ω		
	Tillabéri			α Ω	
	Zinder	α β Ω	α Ω	α β	α β Ω
Women	Maradi	α β Ω			
	Tahoua		α β Ω		
	Tillabéri	B	α β Ω	α Ω	
	Zinder	α β Ω	α Ω	α β Ω	α β Ω

Statistically significant difference in protein intake= α, fat intake= β, carbohydrate intake= Ω
